# Understanding the Impact of Environmentally Specific Servant Leadership on Employees’ Pro-Environmental Behaviors in the Workplace: Based on the Proactive Motivation Model

**DOI:** 10.3390/ijerph20010567

**Published:** 2022-12-29

**Authors:** Baolong Yuan, Jingyu Li

**Affiliations:** School of Business, Central South University of Forestry and Technology, Changsha 410004, China

**Keywords:** environmentally specific servant leadership, workplace pro-environmental behaviors, green self-efficacy, green organizational identity, environmental passion, green shared vision

## Abstract

The increasingly severe environmental situation has brought challenges to the world, and organizations are aware of the importance of environmental management and are committed to changing individuals’ environmental behavior patterns. Based on the proactive motivation model, this study constructs a moderated multiple mediation model to explore the impact mechanism and boundary conditions between environmentally specific servant leadership (ESSL) and employees’ workplace pro-environmental behaviors (WPB). This study examines 440 Chinese industrial sector employees, and the linear regression method test results show that: (1) ESSL significantly promotes employees’ WPB. (2) Green self-efficacy (GSE), green organizational identity (GOI), and environmental passion (EP) act as multiple mediators between ESSL and employees’ WPB. (3) Green shared vision (GSV) positively moderates the relationship between ESSL and employees’ WPB. This study provides a new theoretical perspective on how ESSL affects employees’ WPB, which is analyzed from three aspects: “can do”, “reason to”, and “energized to”. This new mechanism reveals that leaders should transform their leadership style to that of environmental service, and focus on enhancing the employees’ GSE, GOI, and EP. Moreover, ESSL should make copious use of green strategy tools, such as GSV, to realize the above mechanism.

## 1. Introduction

In recent years, environmental issues, such as climate change, rising sea level, and extinction of species have become ever more serious. The public has clearly realized the severity of the environmental crisis, the consequences of environmental degradation, and the threats of climate change. Therefore, countries around the globe have begun to reduce carbon emissions, prevent global warming, strive to protect the ecological environment, and achieve environmentally sustainable development [1]. To address these issues, the 26th and 55th Conference of the Parties of the “UN 2030 Agenda” and the “UN Climate Change Agenda” called for a reduction in carbon emissions to reduce environmental pressures [2]. Environmental issues force companies to mobilize resources and capabilities to adopt environmental management and transition to sustainable green development [3,4]. Research shows, under corporate environmental management, organizational citizenship behavior consistent with environmental management policies is beneficial to pro-environmental results. Therefore, organizations have begun to work on changing the behavior patterns of individuals related to the environment, that is, the pro-environmental behaviors of individuals [3,5].

Pro-environmental behaviors, which are behaviors that consciously seek to minimize the negative impact of an individual’s actions on the natural and constructed world, can be an effective way to achieve workplace sustainability programs [6,7]. They are divided into two aspects according to their background: pro-environmental behaviors in natural environments and pro-environmental behaviors in social organizations. Pro-environmental behaviors in the natural environment refers to the occurrence of crises, such as ecological and environmental disasters, which affect individuals’ pro-environmental behavior by changing their environmental attitudes or motivations. For example, the COVID-19 crisis increased an individual’s sense of urgency in regard to environmental issues through the threat of disease, further strengthening their environmental motivation [8,9]. Pro-environmental behaviors in social organizations mainly include the pro-environmental behaviors of the public, workplace employees, and school teachers and students, which are mainly divided according to the individuals’ role in their social organizations [10,11,12,13,14]. Among them, workplace pro-environmental behaviors can promote sustainable management of the organization’s environment and reduce the economic threat posed by environmental degradation to the organization [15].

The factors that affect employees’ workplace pro-environmental behaviors (WPB) mainly include individual employee factors such as employee values [16,17], attitudes and beliefs [18], and emotions [19], and external situational factors such as interpersonal [15,20], team [15], and organization [21,22,23,24]. Researchers have examined interpersonal situational factors, with leadership as the core, and found that it can affect employee green behavior [25]. Specific styles of leadership can affect employee pro-environmental intentions and behaviors by stimulating employees to develop self-identity [26]. However, existing research mainly focuses on environmental transformational leadership [5,25,27,28], servant leadership [29], empowering leadership [30], responsible leadership [17], and green inclusive leadership [31]. Few have explored the impact mechanism of environmentally specific servant leadership (ESSL) on employee’s WPB.

Based on the servant leadership theory, environmentally specific servant leadership (ESSL) is a leadership style that amplifies the pursuit of environmental protection values, which is environmentally motivated and encourages and serves employees to pursue corporate environmental goals [32,33]. As scholars continue to examine ESSL, its impact on the target environment is gradually becoming clearer, which is concentrated at the organizational and individual levels [34]. The organizational level revolves around the overall environmental performance of the team and the organization [35,36]. The individual level focuses on individual green performance, and green creativity [2,33,36]. Therefore, there are few studies that examine ESSL and employees’ WPB, and the influencing mechanism needs to be further explored.

With the exploration of employees’ green behavior, organizational behavior variables, such as self-efficacy, organizational identity, and emotion have also been incorporated into the green management field, which can stimulate employee behavior by influencing employee motivation [26]. Among them, green self-efficacy (GSE) refers to an individual’s belief in achieving environmental plans and goals [26]. Green organizational identity (GOI) refers to the degree of employees’ internalization of the organization’s environmental values and goals [37]. Environmental passion (EP) refers to employees’ positive emotions to participate in environmental behaviors [38]. Research shows that GSE, GOI, and EP play a positive role in stimulating employees’ creativity and influencing employees’ behavior [39,40]. However, whether these factors can be used as a psychological bridge between ESSL and employees’ WPB is worth further discussion.

Scholars have explored and introduced shared vision, which can guide members toward organizational goals [41], into the field of green environmental management [42]. Green shared vision (GSV) refers to the collective environmental goals with clear and common strategic direction that organizational members have internalized [39]. Although the transformation of leadership style is the main factor for organizations to arouse the enthusiasm of employees in the aspect of pro-environmental behaviors, it is necessary to establish a good GSV to enhance and maintain continuous effort and enthusiasm, that is, continuing to inspire the employees’ GSE, GOI, and EP. Therefore, this study explores the moderating effect of GSV on the relationship between ESSL and WPB from the perspective of vision motivation.

Based on the above, this study aims to address the following questions: (1) Can ESSL affect employee’s WPB in the industrial sector? (2) Can GSE, GOI, and EP play multiple mediating roles in the relationship between ESSL and WPB? (3) Can GSV function as a boundary condition to moderate the overall pathway mechanism of ESSL to WPB? Focusing on the above questions and based on the proactive motivation model, this study examines Chinese industrial sector employees, and discusses the influencing mechanism of ESSL on WPB under the influence of multiple mediators of GSE, GOI, and EP. In general, this study discusses environmental management issues from the micro subject of employees, which can provide guidance to the establishment of the mechanism of national environment protection. Specifically, this study provides a new theoretical explanation for the mechanism that induce employees’ pro-environment behaviors, and profoundly reveals the psychological mechanism between leadership style and WPB. Moreover, the conclusions can help leaders build a management system to guide employees to implement pro-environment behaviors from the dimensions of “can do”, “reason to”, and “energized to”.

This study contributes to the existing research with the following: First, this study constructs the theoretical model of the relationship between ESSL and WPB from the novel perspectives of proactive motivation model. Previous studies rarely involved all types of behaviors such as obligatory or mandatory behaviors, especially voluntary pro-environmental behaviors of individual employees [23]. Starting from the individual initiative of employees, this study uses the active motivation model to reveal the reasons why employees adopt proactive pro-environmental behaviors in the workplace from the perspective of employees’ psychological motivation and verifies the positive promotion effect of ESSL on employees’ proactive WPB.

Second, this study contributes to the multiple mediation model that affects WPB by examining how the proactive motivation model influences employees’ proactive behavior in terms of capability, reasoning, and motivation. Existing studies have explored the influence of organizational climate, responsible leadership, corporate social responsibility, and Green HRM on WPB [7,16,22,25,43,44], but few reveal the mechanism between ESSL and WPB from the perspective of employee motivation, which is not conducive to guiding employees to maintain continuous positive pro-environmental behaviors. Therefore, this study provides a new theoretical perspective to explain how GSE, GOI, and EP respectively act as the capability, reasoning, and motivation that bridges ESSL and WPB.

Last, by considering how GSV constitutes the boundary conditions of the ESSL-to-WPB pathway, this study advances the organizational context-level understanding of the relationship between leadership and employee behavior from the perspective of resource and expectations. How can ESSL more effectively motivate employees to implement pro-environmental behaviors and maintain lasting initiative? This question has not been answered in the existing literature. Organizations are more desirable for employees to produce continuous and active pro-environment behaviors under the influence of ESSL, rather than intermittent ones. Based on this consideration, this paper tries to take GSV as a key and leading factor to strengthen the relationship between ESSL and WPB, hoping to guide employees to continuously release the motivation of WPB. Therefore, this study provides new insights into how GSV, as an organizational context variable, acts as a boundary moderator on the relationship between ESSL and WPB.

## 2. Literature Review and Hypotheses

### 2.1. Proactive Motivation Model

This study uses the proactive motivation model to construct a theoretical analysis framework to examine the relationship between environmentally specific servant leadership (ESSL) and workplace pro-environmental behavior (WPB). The key idea is to clarify the entire goal-driven process and divide the proactive behavior into two stages: proactive goal generation and proactive goal striving [45,46]. Among them, proactive goal generation refers to employees setting goals and planning activities according to their own wishes before accepting tasks; proactive goal striving refers to the behavior and psychological mechanism where employees purposefully seek to achieve proactive goals [45]. The proactive motivation model points out that situational factors, such as individual differences of employees and work environment, can affect the three motivational states of “can do”, “reason to”, and “energized to” in employees, thereby prompting them to generate and strive for proactive goals [45,47]. ESSL can provide employees with environment-related resources and services. Based on the operating mechanism of the proactive motivation model, ESSL is used as a work situation variable that affects employees’ proactive behavior, which may stimulate employees’ three motivational states and prompt employees to adopt WPB [48,49].

### 2.2. Workplace Pro-Environmental Behavior

In a broad sense, pro-environmental behaviors are sustainable behaviors that people engage in their natural environment and attempt to reduce the negative impact of their activities on the environment [50]. In a narrow sense, pro-environmental behaviors are behaviors that individuals in a specific field undertake from an organizational or personal perspective to help the environment, such as recycling paper or saving water at work [40]. Workplace pro-environmental behavior (WPB) refers to the environmental protection behaviors that employees undertake that promote the sustainable environment of an organization, reflecting the willingness of employees to participate in pro-environmental activities [22].

According to the role positioning of employees in an organization, some studies divide employees’ pro-environmental behaviors into in-role pro-environmental behaviors and extra-role pro-environmental behaviors [16]. In-role pro-environmental behaviors are pro-environmental behaviors that employees must implement in the workplace in accordance with job requirements, rules, and regulations, and are related to their work tasks, which are mandatory and guiding [21,51]. Extra-role pro-environmental behaviors are environmental behaviors voluntarily taken by employees, which are not subjected to the formal rules and regulations of the organization [21,52]. The proactive behavior defined by the proactive motivation model is not limited to simple in-role or extra-role behaviors, but measures whether behavior is expected, planned, and exerted through effort [45,53]. There are few studies that examine the influencing mechanism of WPB on proactiveness, therefore, this study starts from the perspective of employee proactiveness to explore the influencing mechanism of WPB.

Based on existing research, the influencing factors of WPB are mainly divided into two factors: individual employee and external situation.

1. Individual employee factors. Employees’ values [17,54], attitude and beliefs [18], emotions [19], and motivation [55] significantly affected WPB. The values of employees determine their environmental attitudes and beliefs and other personal cognitive factors, which in turn guide employees to change their thinking, and promote the generation of controlled and autonomous environmental protection motives, so as to be restrained by pressure or motivated to participate in pro-environmental behaviors [56,57].
2. External situational factors. The external contextual influencing factors of WPB are mainly divided into interpersonal [15,20], team [15], and organizational [21]. First, for the interpersonal level, WPB is reinforced by interpersonal dynamics among leaders and colleagues, including leaders imposing expectations on employees and rolling out green initiatives, open discussions of environmental sustainability among colleagues, and sharing of environmental knowledge to shape workplace interactions [15,20,27,58]. Second, for the team level, team norms and team green work atmosphere will transform corporate environmental protection policies into behavioral norms that reward and support employees and motivate employees to adopt pro-environmental behaviors [15,58]. Third, for the organizational level, corporate green human resource management and social responsibility provide employees with support situations including environmental knowledge and resources, so that employees can generate and strengthen environmental motivation and adopt pro-environmental behaviors [3,28,59,60]. In addition, the rules, regulations, and job descriptions of the enterprise restrict employees’ behaviors in the form of mandatory tasks, while the supervisor support and green internal culture in the organization will help improve employees’ autonomy. These constraints in the external context of the organization will affect employees’ beliefs and significantly influence their pro-environmental behaviors [23,24].

### 2.3. Environmentally Specific Servant Leadership and Workplace Pro-Environmental Behavior

#### 2.3.1. Environmentally Specific Servant Leadership

The effective implementation of various environmental protection actions of organizations depends on their leaders, and the improvement of organizational and individual performance of employees is also closely related to the leaders. Therefore, scholars have started to turn their research focus of corporate development towards environmentally specific servant leadership (ESSL) [61]. ESSL is a servant leadership style, performed by exercising exemplary leadership roles that encourages and serves others to drive organizational and individual environment performance [33,35]. The concept of ESSL includes three key elements: (1) Environmental value incentives. ESSL guides employees to form an environmental value identity through leadership example, and guides employees to adopt environmental behavior through instructions and value incentives [33]. (2) Environmental sustainability. ESSL promotes employees to adopt environmental behaviors through resource services and behavior guidance, with the ultimate goal of promoting the environmental sustainability of organizations and individuals [2]. (3) Result effectiveness. ESSL promotes team and individual green performance through direct or indirect action to bring effective results to the organization and employees [36].

There is some overlap and distinction between ESSL and other leadership types. ESSL shares the same traits as servant leadership, both prioritize the needs of employees and are accountable to the organization and its stakeholders [35]. However, ESSL focuses the servant leadership behavior on green management, adopts pro-environmental leadership practices, and encourages employees to be motivated to pursue environmental goals [33]. Similar to other environment-oriented leaderships, ESSL provides employees with environmentally oriented resources, motivates employees’ environmental mission and guides them to adopt environmental-friendly behaviors, thereby improving the organization’s environmental performance. However, in contrast to environmental transformational leadership, which is motivated to pursue organizational environmental goals, ESSL is motivated to meet employee needs and follow an other-oriented value system to guide employees toward shared goals [33]. Environmentally specific charismatic leadership focuses on motivating employees’ environmental mission through the charisma of the leader, so that employees can collectively identify with environmental goals, while ESSL focuses on providing employees with green-related resources and services to encourage employees to contribute to the sustainable development of the organization [33,62].

The little research on ESSL have mostly focused on the hotel service sector and mainly examined organization and individual employees. From an organizational perspective, ESSL can significantly affect the overall environmental performance of teams and organizations [35,36]. ESSL is compatible with altruism and collectivism culture and enables employees to form green norms by providing green-related resources and guidance. In a green atmosphere, team members influence each other and improve team green performance [35]. From the perspective of individual employees, on the one hand, ESSL provides employees with green-related resources through the implementation of green crafting. Employees can rely on proactive resource acquisition to improve individual environmental performance [2,36,63]. On the other hand, ESSL will promote employees to generate green climate in the organization through example and demonstration. Under ESSL influence, employees integrate leader value incentives, resource services, and seek innovative solutions to stimulate green creativity [64,65].

#### 2.3.2. The Relationship between Environmentally Specific Servant Leadership and Workplace Pro-Environmental Behavior

This study analyzes the relationship between ESSL and WPB from the two stages of employee proactive behaviors, including proactive goal setting and proactive goal striving, and explains the formation principles of the two stages from the goals, cognitive and behavioral, and resources perspectives.

First, the proactive motivation model explains the relationship between ESSL and WPB from a goal perspective. (1) ESSL is relatively clear on the organization’s environmental protection goals and implements corresponding environmental values and environmental behaviors. At the same time, ESSL encourages employees to participate in environmental actions and retain their freedom to deal with environmental issues, which helps employees to set clear proactive environmental goals [66,67]. (2) The proactive motivation model proposes that leaders can influence employees’ motivational states and drive their goal setting and striving. ESSL promotes the achievement of personal goals by serving and supporting employees to generate intrinsic motivations, such as self-efficacy or identification [45,46].

Second, the social learning theory explains the relationship between ESSL and WPB from a cognitive and behavioral perspective. The social learning theory states that behavior is the result of the interaction of individual cognition and the environment, and employees can act by imitating the values and behaviors of role models [68]. (1) ESSL conveys workplace expectations to employees through a consistent environmental behavior model, provides employees with environmental protection action directions and authorization, and motivates employees to actively set and strive for environmental protection goals [31,40,50,63]. (2) ESSL advocates environmental protection values and exhibits the characteristics of environmental protection behavior that make employees regard it as a role model for behavior imitation, and under its leadership, employees are more inclined to adopt environmental protection behaviors to achieve environmental protection goals [33,63,66,69].

Last, the conservation of resources theory explains the relationship between ESSL and WPB from a resource perspective. In the process of goal striving, employees often adopt proactive resource strategies, take proactive behaviors, and gain benefits by actively seeking and investing resources [35]. As the conservation of resources theory points out, employees will actively seek and accumulate additional resources, and invest them on expected behaviors through the spiral accumulation of resources [70,71]. ESSL can provide employees with green knowledge, skills, and other related resources to meet the needs of employees to achieve their proactive goals, therefore, they invest resources in green achievements, such as pro-environmental behaviors [43,54,64,72]. Based on the above, this study proposes the following hypothesis:

**Hypothesis 1** **(H1).** *Environmentally specific servant leadership is positively related to employees’ workplace pro-environmental behavior.*

### 2.4. “Can Do” and Green Self-Efficacy as a Mediator

#### 2.4.1. Green Self-Efficacy

Green self-efficacy (GSE) is the confidence and self-judgment of employees to take environmental protection actions before completing their goals. It refers to employees’ belief in their ability to organize and implement action plans to achieve environmental goals [26]. From the perspective of proactive motivation model, GSE represents employees’ confidence in achieving environmental goals and is related to goal setting. High GSE is conducive to employees’ proactiveness to set goals and is used as a source of employees’ “can do” motivation to analyze its relationship with environmentally specific servant leadership (ESSL) and employees’ workplace pro-environmental behavior (WPB) [26,45].

#### 2.4.2. Environmentally Specific Servant Leadership and Green Self-Efficacy

Self-efficacy refers to employees’ belief in their ability to organize and execute actions and is influenced by different leadership styles in the action process. GSE is derived from self-efficacy, which is also affected by leadership [73]. This study explores the relationship between GSE and ESSL from the perspectives of resources, cognition, and self-determination. First, according to the conservation of resources theory [70,71], ESSL can train employee skills and provide environmental protection related resources. On the premise of obtaining sufficient environmental resources, employees will show strong beliefs, which will improve GSE [26,74]. After employees obtain environmental resources, they will seek feedback to support their environmental protection beliefs, and ESSL will improve employees’ GSE through frequent positive feedbacks [45]. Second, according to the social cognitive theory, ESSL is a practitioner of organizational environmental management. ESSL provides goals and directions for employees’ environmental behavior by caring about employee environmental protection initiatives and helping employees practice environmental protection actions, which makes employees believe that they have the ability to overcome difficulties faced in achieving environmental goals, which will improve GSE [39,75,76,77]. Last, based on the self-determination theory, which focuses on the degree to which an individual’s behavior is self-motivated and self-determined, ESSL gives employees the freedom to deal with environmental issues, provides inclusive support and encouragement for employees’ environmental behavior, internalizes employees’ social expectations, and further stimulates employees’ autonomy and intrinsic motivation, so that they have the belief to firmly complete environmental tasks [66,78,79].

#### 2.4.3. Green Self-Efficacy and Workplace Pro-Environmental Behavior

GSE is an integral part of employees’ individual beliefs, and as an intrinsic motivation, beliefs can provide employees with action directions and guarantees, which makes GSE a key psychological motivation factor that drives employees to adopt environmental protection behaviors [26]. This study further explores the relationship between GSE and WPB.

First, the proactive motivation model proposes that individuals’ beliefs, in regard to success in a particular field, will affect their goal setting through behavioral risk assessments, and that goal achievement and feedback-seeking behavior is also affected by personal risk assessments, which show the importance of personal beliefs in shaping proactive actions [45,47,53]. From the perspective of employee behavior, self-efficacy can enhance perseverance and improve employees’ willingness to overcome obstacles, making them feel that they can control the development of the situation and have a certain impact on the results, which satisfies the conditions for proactive behavior and confirms the impact of GSE on WPB [45]. Second, a high level of GSE also helps employees to strive for proactive goals [26,79]. In addition, high self-confidence will drive individuals to take proactive actions, where GSE can shape internal motivation by enhancing their perception of their own environmental capabilities and promote employees to adopt pro-environmental behaviors [73,74,80]. Last, employees with high GSE are more confident in their own abilities and set challenging and reasonable goals, which provides employees with stronger internal motivation and gives them strong determination to perform better in the process of striving for environmental goals, which will result in the adaptation of pro-environmental behavior [74,81,82].

#### 2.4.4. The Mediating Role of Green Self-Efficacy

Based on the above, this study further proposes that GSE plays an important mediating role in the influence of ESSL on WPB. ESSL can improve employees’ beliefs by providing training and other environment-related resources. Employees believe that they can achieve their set environmental goals through the resources they receive. While improving GSE, they can further identify their own environmental capabilities and build a high degree of self-confidence, which provides employees with internal motivation and promote pro-environmental behavior [69,74]. In addition, ESSL can voluntarily commit resources and adopt pro-environmental behaviors by directing employee environmental behaviors to make them feel that they have sufficient control over the development of the situation and the ability to achieve environmental-related milestones [47,73]. Based on the above, this study proposes the following hypothesis:

**Hypothesis 2** **(H2).** *Green self-efficacy mediates the relationship between environmentally specific servant leadership and employees’ workplace pro-environmental behavior.*

### 2.5. “Reason to” and Green Organizational Identity as a Mediator

#### 2.5.1. Green Organizational Identity

Based on corporate green management and competition, green organizational identity (GOI) originates from organizational identity and is an organizational identity model in the environmental field. GOI specifically refers to an explanatory plan for environmental management and protection of an organization jointly constructed by its members, which reflects the degree of internalization of employees’ commitment to the organization’s environmental values and goals [37]. From the perspective of the proactive motivation model, when goals set by employees are reinforced through internal and external rules, it will cause the employees to have a reason to complete the goals, that is, the “reason to” motivation of employees to take proactive behaviors. The GOI of employees reflects their role and values, clearly defines their responsibilities and missions for their actions, and promotes them to adopt pro-environmental behaviors [45]. Therefore, this study uses GOI as the source of employee motivation to analyze its relationship with environmentally specific servant leadership (ESSL) and workplace pro-environmental behavior (WPB).

#### 2.5.2. Environmentally Specific Servant Leadership and Green Organizational Identity

ESSL can convey organizational norms and environmental values to employees through behavioral demonstrations and promote employees to form environmental awareness consistent with the organization, thus improving employees’ GOI. This process can be further analyzed from three perspectives.

First, the leadership characteristics of ESSL. (1) Leadership can enhance employees’ sense of belonging, reduce uncertainty, satisfy employees’ self-needs, and enhance organizational identity [83]. (2) ESSL can convey green information to employees and take pride in participating in green responsibility behaviors through organizational green strategies, to meet the psychological needs of employees, make employees feel the charm of the organization, and find a suitable match between the organization and the individual [36,84,85]. Second, ESSL can guide employees to generate environmental role orientation based on leadership role identity, and form GOI [26]. Last, based on the value resources perspective, ESSL puts environmental interests at the top of its actions, emphasizing contributions to improve the environment to transmit environmental values, which can be used as an environmental resource to promote the internalization of employees’ environmental values, thereby forming GOI [86,87].

#### 2.5.3. Green Organizational Identity and Workplace Pro-Environmental Behavior

GOI enables employees to recognize their environmental role in the organization’s environmental initiatives, understand and agree with the environmental goals set by the organization, and give a deeper meaning to environmental behavior, which is conducive to employees’ adoption of WPB [88,89]. Social identity theory can better explain the relationship between GOI and employees’ WPB. The theory suggests that there are two core motives for identifying with a group, namely the need for “self-categorization” and the need for “self-enhancement”. The former pertains to the increased safety and the reduction of uncertainty that collective identification offers to employees, whereas the latter is concerned with the enhancement of their sense of collective self-esteem [43,90]. Based on this theory, we explore the relationship between GOI and WPB as follows.

First, from the perspective of employee role identification, employees with high GOI regard themselves as part of the organization and identify with their environmental-friendly role positioning, and actively seek to solve organizational environmental problems and make environmental-friendly behaviors [60,79,91]. With high GOI, employees are more inclined to cautiously control relevant resources to make decisions for green actions and meet expectations for environmental protection roles through environmental protection actions [92]. Second, from the perspective of social interaction, employees with GOI see themselves as subordinates of the organization, deal with environmental issues with a positive attitude, and utilize the ownership of environmental identities in the organization, which means the employee’s GOI can act as an intrinsic motivational force to influence pro-environmental behaviors [88]. Conversely, an individual’s identification with the organization can enable them to make contributions to the organization. Under the influence of GOI, employees are motivated to think about environmental issues, form new cognitions, and think about the next action direction, which is beneficial to the adoption of pro-environmental behavior under the influence of the organizational environment [93].

#### 2.5.4. The Mediating Role of Green Organizational Identity

Based on the above, this study proposes that GOI plays an important mediating role in the relationship between ESSL and WPB. Leaders are important promoters of corporate green behaviors. When companies face environmental pressure, leaders are the first to bring environmental protection actions into the focus of the organization and use the example of leaders to drive and enhance employee pride to influence employees to form GOIs. Employees with high GOIs will seek to discover green technologies and apply their knowledge to solve corporate environmental problems [88]. In addition, ESSL is the maker of the organization’s environmental policy and can provide employees with environmental protection resources and encourage employees to contribute to the environment by integrating environmental resources. This material and spiritual encouragement enables employees to generate GOI for the organization’s environmental protection practices. Employees are guided by GOI beliefs to reflect on their membership and to adopt environmental behaviors [93,94]. Based on the above, this study proposes the following hypothesis:

**Hypothesis 3** **(H3).** *Green organizational identity mediates the relationship between environmentally specific servant leadership and employees’ workplace pro-environmental behavior.*

### 2.6. “Energized to” and Environmental Passion as a Mediator

#### 2.6.1. Environmental Passion

Environmental passion (EP) refers to the positive emotions of employees who want to participate in environmental behaviors and is an extension of the harmonious passion theory in the field of environmental sustainability [38]. The proactive motivation model points out that positive emotions can broaden the individual’s ideological reserve, generate action tendencies, and affect the individual to set and effectively strive towards their goals, which can be viewed as a “energized to” motivation for individuals to be proactive [45,95]. As an indicator of intrinsic motivation, EP can make up for the loss of psychological resources caused by employees’ adoption of environmental behaviors and make employees more energetic and motivated to actively participate in pro-environmental behaviors [40]. Therefore, this study believes that EP meets the energy motivation conditions for employees to adopt workplace pro-environmental behavior (WPB).

#### 2.6.2. Environmentally Specific Servant Leadership and Environmental Passion

Existing studies have revealed the impact mechanism of different leadership styles on EP [40,96]. Based on the commonalities of leaders, this study preliminarily speculates that environmentally specific servant leadership (ESSL) will affect employee EP, which is analyzed from two perspectives. From the perspective of the affective events theory [97], in the process of direct interaction between direct leaders and subordinates, environmentally specific servant leadership behaviors that pay attention to environmental issues can be regarded as specific affective events, which are crucial to stimulate the harmonious environmental passion of subordinates [40]. First, ESSL adopts environmental protection behaviors and conveys the determination and attitude of green management through environmental protection demonstrations. Employees imitate and learn from ESSL’s environmental behaviors and attitudes, thereby motivating EP [64]. In addition, ESSL will make environmental commitments, articulate environmental vision, and associate it with employees’ personal identities, strengthening the connection between employees and the organization, and further stimulate employee EP [38]. Second, ESSL can motivate employees to achieve their preset environmental protection goals by encouraging employees to contribute to environmental protection. The environmental contributions made by employees will generate optimism and stimulate EP [38]. Third, ESSL will account for employees’ environmental protection initiatives and provide help for their environmental protection actions, and personalized care and guidance from leaders will provide employees with emotional support and generate EP [40].

In addition to affective events, EPs are also influenced by interpersonal chains. Enthusiasm for the environment can be transmitted through inter-individual or inter-group chains, forming emotional contagion [98]. ESSL can give employees a common emotion of environmental protection through emotion transmissions and participate in pro-environmental behavior under the guidance of their behavior and values [96].

#### 2.6.3. Environmental Passion and Workplace Pro-Environmental Behavior

The emotional state of an individual can activate their psychological motivation, enabling the individual to energetically participate in the target activity, and at the same time, be more inclined to devote their energy to pleasurable activities [99]. Therefore, this study believes that employees’ EP is a positive emotion, which can bring a sense of satisfaction and happiness and affect employees’ actual behavior and promote active participation in the pro-environmental behavior that the organization respects [38,100]. Research has confirmed that an employee’s EP focus expands from the self to the environment and drives employee adoption of WPB [100,101]. In addition, EP, a positive emotional experience, can motivate behavior, motivate employees to participate in challenging activities, and substitute it into the field of organizational environmental management, which can promote employees to adopt WPB [40].

#### 2.6.4. The Mediating Role of Environmental Passion

In summary, ESSL can provide support and help employees by interacting with them, and its environmental attitude and commitment can strengthen the connection between employees and the organization, spread environmental values, and inspire EP [64]. At the same time, employees are emotionally transmitted and driven to generate EP through the interpersonal chain [98]. The positive emotions of EP generated by employees under the influence of ESSL can bring satisfaction to employees and motivate employees to actively participate in environmental protection behaviors, and the emotional experience of participating in activities can further motivate them to participate in challenging activities, which promotes WPB [40,102]. Based on the above, this study proposes the following hypothesis:

**Hypothesis 4** **(H4).** *Environmental passion mediates the relationship between environmentally specific servant leadership and employees’ workplace pro-environmental behavior.*

### 2.7. The Moderating Role of Green Shared Vision

Based on the environmental era, Green shared vision (GSV) is a green concept proposed by Chen and Chang [39], which refers to the collective environmental goals and clear and common strategic directions that have been internalized by organizational members. Organizations with high GSV can help employees build an organization-centric development blueprint, motivate employees to exceed expected goals based on organizational norms and values, and serve as a resource to guide employees to formulate development strategies [39,103]. GSV also affects the strength of ESSL’s influence on WPB’s capability, reasoning, and motivation.

First, the vision can reflect the goals of the organization and help employees determine the focus and direction of action. Under high GSV influence, ESSL can provide more targeted action plans and resource support according to employees’ environmental goals. The material and spiritual incentives from leaders are strengthened in the atmosphere of the organization’s pursuit of environmental sustainability, and employees’ belief are strengthened to achieve environmental goals, which improves GSE [63,104].

Second, employees in high GSV organizations are more inclined to make emotional commitments to the organization under the guidance of ESSL and are more likely to understand the organization’s environmental goals and values. The influence of the leadership on it will be stronger, and the employees are more inclined to accept the leadership’s exemplary role, regard themselves as part of the organization, and enhance their own GOI to promote the common development of the organization and individuals [44,105].

Last, according to the expectancy theory, under the influence of high GSV, employees expect the organization to maintain environmental spiritual incentives. At this time, the environmental protection values and environmental incentives advocated by ESSL further meet the expectations of employees, so they can make positive emotions and work feedback [103]. In addition, under the guidance of GSV, ESSL’s interaction with employees will deepen their understanding of environmental goals and realize that their environmental contributions are meaningful, thereby enhancing EP [40]. Based on the above, this study proposes the following hypothesis:

**Hypothesis 5** **(H5).** *Green shared vision mediates the relationship between environmentally specific servant leadership and green self-efficacy.*

**Hypothesis 6** **(H6).** *Green shared vision mediates the relationship between environmentally specific servant leadership and green organizational identity.*

**Hypothesis 7** **(H7).** *Green shared vision mediates the relationship between environmentally specific servant leadership and environmental passion.*

**Figure 1:**
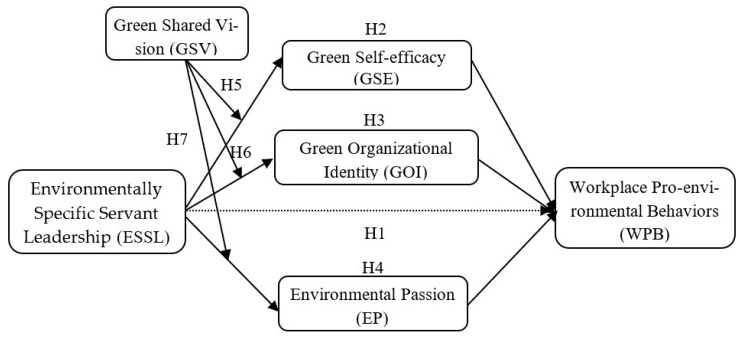
Theoretical model.

In summary, the theoretical model of this study is shown in Figure 1.

## 3. Research Methodology

### 3.1. Sample Description

The variables involved in this study, such as green self-efficacy, green organizational identity, and environmental passion, belong to the individuals’ psychological perception and are difficult to be measured with second-hand observational data. Therefore, this study used questionnaires to collect first-hand data. We emphasized in the preface section of the questionnaire that is only used for academic research, the respondents should fill it out voluntarily, there are no right or wrong answers, and the content of the questionnaire is strictly confidential. This study examines the employees of Chinese industrial firms. The official survey time is from July 2021 to December 2021. A total of 526 questionnaires were distributed through Credamo, a professional survey platform (Credamo is a professional research and modeling integrated data platform independently developed by Beijing Easy Digital Modeling Technology Co., LTD. It is committed to providing one-stop solutions for large-scale research, data collection, modeling analysis and commercial application for scientific research institutions, enterprises, and individuals). After excluding invalid questionnaires, a total of 440 valid questionnaires were recovered with an effective recovery rate of 83.65%. The sample description statistics of the questionnaire are listed in Table 1.

### 3.2. Variable Measurement

Scales used in existing research are used as measurement tools in this study. All variables are measured using a five-point Likert scale, where 1 to 5 respectively represent “strongly disagree”, “disagree”, “undecided”, “agree”, and “strongly agree”. The specific assessment scale is as follows (Appendix A):

1. Environmentally specific servant leadership (ESSL): The measurement items refer to the Environmental Service Leadership Scale adapted by Luu [66], which is directly scored by employees to their superiors, with a total of 12 items. Example items include “My supervisor cares about my eco-initiatives” and “My supervisor emphasizes the importance of contributing to the environmental improvement”.
2. Workplace pro-environmental behaviors (WPB): The measurement items refer to the Employee Pro-environmental Behavior Scale of Robertson and Barling [38], with a total of 7 items. Example items include “I print double sided whenever possible” and “I put compostable items in the compost bin”.
3. Green self-efficacy (GSE): The measurement items refer to the Green Self-Efficacy Scale of Chen and Chang [26], with a total of 6 items. Example items include “We feel we can succeed in accomplishing environmental ideas” and “We can achieve most of environmental goals”.
4. Green organizational identity (GOI): The measurement items refer to the Green Organizational Identity Scale of Chen [37], with a total of 6 items. Example items include “The company’s top managers, middle managers, and employees have a strong sense of the company’s history about environmental management and protection” and “The company’s top managers, middle managers, and employees have a sense of pride in the company’s environmental goals and missions”.
5. Environmental passion (EP): The measurement items refer to the Environmental Passion Scale of Robertson and Barling [38], with a total of 10 items. Example items include “I am passionate about the environment” and “I enjoy practicing environmental-friendly behaviors”.
6. Green shared vision (GSV): The measurement items refer to the Green Shared Vision Scale of Chen [26], with a total of 4 items. Example items include “There is commonality of environmental goals in the company” and “There is total agreement on the company’s strategic environmental direction”.

According to research on the pro-environmental behaviors of leaders and employees, there may be a certain relationship between employees’ gender, age, tenure, and employee pro-environmental behaviors [65]. In this study, the gender, age, education, and tenure of employees are used as control variables.

### 3.3. Common Method Variance

This study adopts procedural control and statistical control to avoid the common method variance issue. In terms of program control, first, this study collects data nationwide through online methods, and the sample is limited to industrial organizations to ensure the diversity of data sources and measurement environments. Second, this study adjusts the fuzzy items through pre-investigation, and adopts an anonymous filling method to ensure the authenticity of the questionnaires. In terms of statistical control, principal component analysis was performed using Harman’s univariate test. The results showed that the first principal component explained only 45.87% of the variance, which is less than 50%, indicating that no single factor explained a majority of variance, so there was no serious common method variance issue.

## 4. Empirical Results

### 4.1. Reliability and Validity

The Cronbach’s α of each variable in this study is greater than 0.7, and the CICT is greater than 0.5 (Table 2), indicating good reliability of the scales. This study uses the AMOS software for confirmatory factor analysis, χ^2^/df = 1.947 < 3.000, RMSEA = 0.046 < 0.080, CFI = 0.937 > 0.900, IFI = 0.937 > 0.900, which shows good model fitting. Table 2 shows that the factor loading of the items has a minimum value of 0.637 and a maximum value of 0.837, which are both higher than the threshold of 0.5, indicating high reliability. The CR values were all greater than 0.8 and higher than the threshold of 0.6, indicating that each variable is homogeneous, internally consistent, and reliable. The average variance extracted (AVE) value of each variable is greater than 0.5, indicating that the scale has good convergent validity, and the correlation coefficient between each factor is less than the square root of AVE, indicating that the scale has good discriminant validity.

### 4.2. Descriptive Statistics Analysis

Table 3 reports the mean, standard deviation, and correlation coefficient of each variable. It can be seen that the correlation of each variable is significant, and the research hypotheses are supported. Multicollinearity test results show that the maximum value of VIF in each regression model was 2.158 (<5), indicating no serious multicollinearity issue.

### 4.3. Hypothesis Testing

#### 4.3.1. Principal Effect Testing

On the basis of controlling for the gender, age, educational background, and working years of employees, this study uses the hierarchical regression analysis method to test the hypothesis [44,79]. The test results are shown in Table 4. Model 8 shows that environmentally specific servant leadership (ESSL) is positively correlated with employees’ workplace pro-environmental behavior (WPB) (*β* = 0.716, *p* < 0.001), supporting H1. This means that when the leadership style changes to environmental protection and provides relevant resources and services to employees, it can better motivate their pro-environment behaviors.

#### 4.3.2. Mediating Effect Testing

Model 2 shows that ESSL is positively correlated with green self-efficacy (GSE) (*β* = 0.719, *p* < 0.001). Model 9 shows that GSE is positively correlated with WPB (*β* = 0.640, *p* < 0.001). Model 12 shows that when ESSL and GSE are introduced into the regression equation at the same time, GSE still significantly promotes WPB (*β* = 0.272, *p* < 0.001), and ESSL still has a significant effect on WPB (*β* = 0.521, *p* < 0.001). Compared with the estimation coefficient of ESSL in Model 8, it is decreased, indicating that GSE plays a partial mediating role, supporting H2. This result indicates that ESSL can encourage employees’ pro-environment behaviors through enhancing their confidence in their ability to deal with workplace environmental issues.

Model 4 shows that ESSL is positively correlated with green organizational identity (GOI) (*β* = 0.691, *p* < 0.001). Model 10 shows that GOI is positively correlated with WPB (*β* = 0.640, *p* < 0.001). Model 13 shows that when ESSL and GOI are introduced into the regression equation at the same time, GOI still significantly promotes WPB (*β* = 0.295, *p* < 0.001), and ESSL still has a significant effect on WPB (*β* = 0.513, *p* < 0.001). Compared with the estimation coefficient of ESSL in Model 8, it is decreased, indicating that GOI plays a partial mediating role, supporting H3. This means that when employees internally identify with the organization’s vision, goals, and significance regarding environmental protection, employees and leaders will reach an internal consistency in terms of pro-environmental behaviors. This recognition will also encourage employees to actively adopt pro-environmental behaviors according to the organization’s requirements or under the influence of leaders.

Model 6 shows that ESSL is positively correlated with environmental passion (EP) (*β* = 0.732, *p* < 0.001). Model 11 shows that EP is positively correlated with WPB (*β* = 0.731, *p* < 0.001). Model 14 shows that when ESSL and EP are introduced into the regression equation at the same time, EP still significantly promotes WPB (*β* = 0.451, *p* < 0.001), and ESSL still has a significant effect on WPB (*β* = 0.386, *p* < 0.001), Compared with the estimation coefficient of ESSL in Model 8, it is decreased, indicating that EP plays a partial mediating role, supporting H4. This shows that ESSL can provide support and help employees by interacting with them, and its environmental attitude, emotion, and commitment can strengthen the connection between employees and the organization, spread environmental values, inspire environmental passion, and eventually transform employees’ EP into WPB.

#### 4.3.3. Moderating Effect Testing

**Figure 2:**
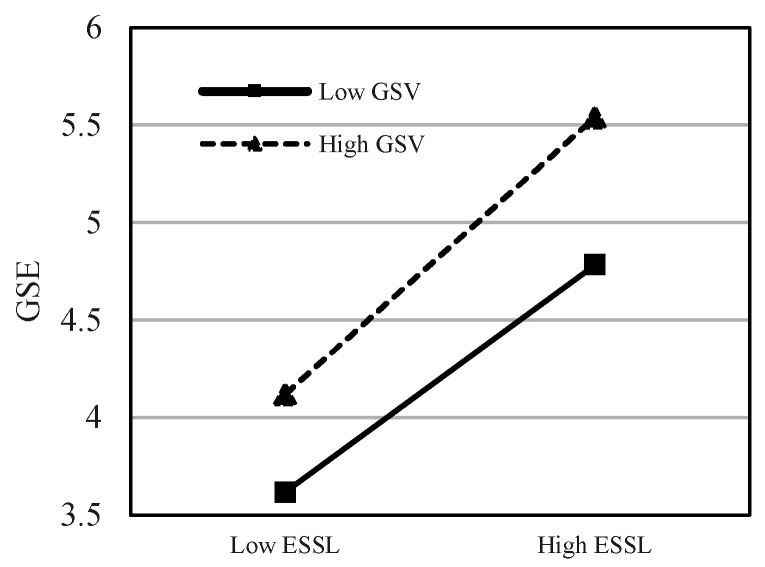
The moderating effect of GSV on the relationship between ESSL and GES.

**Figure 3:**
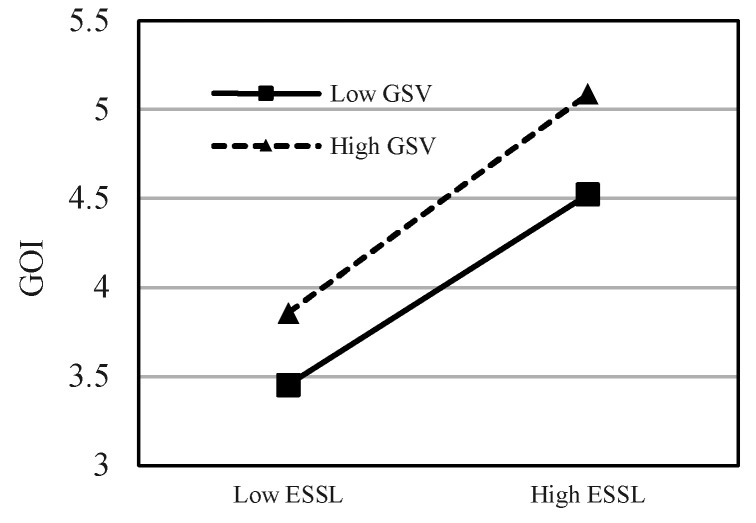
The moderating effect of GSV on the relationship between ESSL and GOI.

**Figure 4:**
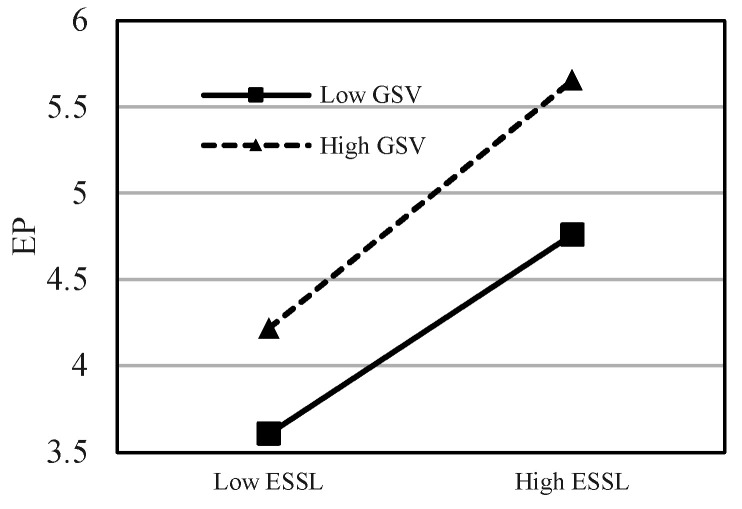
The moderating effect of GSV on the relationship between ESSL and EP.

This study uses the hierarchical regression analysis method to test the moderating effect, introduces the interaction term between ESSL and green shared vision (GSV) to build a model, and examines the significance of the regression coefficient of the interaction term. The moderating effect results of GSV are shown in Table 5, which show that the interaction terms significantly moderate GSE, GOI, and EP (*β* = 0.156, *p* < 0.001; *β* = 0.096, *p* < 0.05; *β* = 0.173, *p* < 0.001). Thus, H5, H6, and H7 are supported. This means that green sharing vision, as an important green strategy tool commonly used by leaders, can help the organization to motivate the psychological motivation of employees’ pro-environment behaviors. In order to further reveal the moderating effects of GSV between ESSL and GSE, GOI, and EP, the moderation effect diagrams are drawn in Figure 2, Figure 3 and Figure 4.

## 5. Discussion

### 5.1. Theoretical Contributions

This study provides new insights into the structural linkages of environmentally specific servant leadership (ESSL) and workplace pro-environmental behavior (WPB) based on the proactive motivation model. The results significantly advance the debate on the mechanism of ESSI and WPB in significant ways.

First, this study verifies that ESSL can significantly promote WPB based on the proactive motivation model. The results show that ESSL can guide employees to set clear goals and provide motivation. Employees are guided by ESSL’s example to generate environmental cognition, and by actively seeking and accumulating relevant resources provided by ESSL, the obtained resources are invested in environmental behaviors, and to finally achieve expected environmental behavior. This conclusion is basically consistent with studies such as Aboramadan et al. [64], Luu [35], and Luu [65], which point out that ESSL plays a positive role in promoting organizational green performance and stimulating employees’ green creativity. This inference is consistent with the viewpoint of the social learning theory and the resource conservation theory. Furthermore, this study obviously and significantly extends the conclusions of Xia et al. [2], Luu [36], Luu [33], Mughal et al. [75], Afsar et al. [43], and Afsar et al. [44]. This study provides a new perspective based on the proactive motivation model and integrated social learning theory and resource conservation theory for the related research of ESSL and WPB and enriches the related literature of ESSL and WPB in the environmental field.

Second, this study finds that green self-efficacy (GSE), green organizational identity (GOI), and environmental passion (EP) play multiple mediating roles between ESSL and WPB. This means that ESSL should focus on enhancing employees’ green self-efficacy, improving their green organizational identity, and stimulating their environmental passion, so as to motivate them to actively implement pro-environmental behaviors. In this process, we fill the research gap of the capability, reasoning, and motivation of employees’ proactive behavior in the proactive motivation model. To some extent, these conclusions are consistent with Chang et al. [103], Chen [37], Chen et al. [26], Li et al. [40], Mughal et al. [75], and Yin et al. [100], and they all point out that GSE, GOI, and EP play an important role in stimulating employees’ behaviors and abilities. These inferences are consistent with the viewpoint of the self-determination theory, the social identity theory, and the affective events theory. Furthermore, this study extends the conclusions of Rabiul et al. [29], Robertson and Barling [38], Tian and Robertson [57], Xia et al. [2], and Zhang et al. [17] in a meaningful way. This study explores the multiple environmental factors that influence the relationship between leaders and employees’ pro-environmental behaviors, verifies the mediating role of GSE, GOI, and EP between ESSL and WPB, and explains that GSE, GOI, and EP function as bridges of capability, reasoning, and motivation that connect ESSL with WPB. Therefore, this study constructs a new theoretical perspective to explain the impact mechanism between ESSL and WPB through the proactive motivation model and deepens the theoretical literature on the impact of ESSL on WPB, helping practitioners to formulate and implement effective environmental management measures.

Third, this study points out that ESSL affects the boundary conditions of WPB through GSE, GOI, and EP. Specifically, this study empirically demonstrates the moderation mechanism of green shared vision (GSV) in the organizational context on leader and employee behavior. The results show that GSV can guide employees to accept the leadership’s environmental protection values and material and spiritual incentives under a shared vision, thereby enhancing their GSE, GOI, and EP. In addition, under the influence of GSV, leadership behavior can better meet employees’ expectations and further promote WPB. The conclusions are consistent with the studies of Afsar et al. [44], Chang et al. [103], and Chen et al. [26], which confirm the positive role of GSV in influencing the attitude, motivation, and behavior of employees. In particular, it helps to enhance the interaction between leaders and employees in the field of environmental management. In the meantime, this paper also extends the conclusions of Afsar et al. [43], Li et al. [40], Luu [33], Mughal et al. [75], Peng et al. [96], and Xia et al. [2], which provides new views and evidence on how to continuously stimulate the employees’ motivation between ESSL and WPB. From the perspective of resources and expectations, this study explores the moderating role of GSV in the impact pathway of ESSL on WPB and expands the related literature on GSV to incorporate it into the field of organizational management as a boundary constraint. Furthermore, this study explores the operation mechanism of GSV as an organizational variable to limit the relationship between ESSL and WPB and explains its boundary moderating effect as a situational variable in the field of organizational management from a new theoretical perspective.

### 5.2. Managerial Implications

This study has the following managerial implications: First, leaders should commit themselves to shape environment-led values, foster environmental goals, and proactively adopt environmental-friendly behaviors. For example, leaders should show the habit or awareness of saving water, electricity, paper, and other resources and energy in the company, and emphasize the importance of energy saving and environmental protection in the workplace when they are with employees. Where possible, leaders can use public transportation, bicycle, or walk to and from work to establish a pro-environment image among employees, thus influencing employees’ awareness and behavior. In addition, leaders should improve employees’ environmental capabilities through training and mentoring. For example, courses and practical training related to environmental protection behaviors should be arranged in new employee training and on-the-job training.

Second, leaders should provide resources to support employees to adopt pro-environmental behaviors. For example, companies can provide double-sided printers, dry and wet garbage separation bins, free reusable lunch boxes, and replace the companies’ utilities with energy-efficient ones for employees. Leaders can also provide material and spiritual rewards to enhance employees’ environmental beliefs, so as to stimulate employees’ ability and motivation. In addition, leaders need to increase employees’ sense of belonging to the organization so that employees have a green identity that matches the organization’s goals and enhance the organization’s sense of identity to promote employees to adopt pro-environmental behaviors. For example, companies can select candidates with pro-environmental awareness in the recruitment and selection process. Last, leaders are encouraged to communicate their environmental enthusiasm through interaction with employees, change employees through emotional transmission, and increase pro-environmental behaviors in the workplace.

Third, the green shared vision in an organization is particularly important to promote the pro-environmental behavior of employees. Companies should increase the propaganda on their vision, so that employees can agree with the vision of sustainable development in the organization and proactively undertake pro-environmental behavior. At the same time, organizations should pay attention to the green concept in the workplace and commit themselves to building a green and sustainable organizational management environment.

### 5.3. Limitations and Future Research

This study has the following three limitations: First, this study examines the employees of Chinese industrial organizations. In the future, the scope of the sample can be extended to other sectors related to green development, such as agriculture, construction, or tourism to improve the universality of this research. Second, this study considers GSE, GOI, and EP as three major motivations that affect WPB. In the future, other motivational variables, such as environmental commitment and organizational support, can be explored and examined. Third, this paper uses GSV, an organizational-level variable, as a moderating factor, in future research, individual-level factors, such as face awareness, can be examined as boundary conditions. Finally, this paper relied on the same source, self-reported data, which raises concerns about the effects of common method variance. In the future, data from both leaders and employees can be used for cross-hierarchy analysis, or third-party observation data can be used to measure employees’ pro-environment behaviors instead of asking them to fill in the questionnaire.

## 6. Conclusions

In the existing literature, the issue of how to continuously and efficiently stimulate employees’ pro-environment motivation through leadership, and then transform it into effective pro-environment behavior is a problem worthy of further exploration. Our study specifically addresses this gap. Based on the proactive motivation model, this study constructs a moderated multiple mediation model to explore the impact mechanism and boundary conditions between environmentally specific servant leadership (ESSL) and employees’ workplace pro-environmental behaviors (WPB). We found that ESSL significantly promotes employees’ WPB. Green self-efficacy (GSE), green organizational identity (GOI), and environmental passion (EP) act as multiple mediators between ESSL and employees’ WPB. Green shared vision (GSV) positively moderates the relationship between ESSL and employees’ WPB. This study also provides important theoretical and managerial implications. Specifically, it provides a new theoretical perspective on how ESSL affects employees’ WPB, which is analyzed from three aspects: “can do”, “reason to”, and “energized to”. This new mechanism can inspire leaders to transform their leadership style to environmental service. The focus of environmental service leaders is to enhance the employees’ GSE, GOI, and EP. Moreover, environmental service leaders should be good at using green strategy tools, such as GSV, to realize the above mechanism. Finally, this study points out its limitations and proposes ideas that can be explored in future research.

## Figures and Tables

**Table 1:** Sample characteristics (N = 440).

Characteristics	Classification	Counts	Frequency (%)
Gender	Female	243	55.2
	Male	197	44.8
Age	Less than 25	34	7.7
	26–35	310	70.5
	36–45	65	14.8
	46 and above	31	7.0
Edu	High, Primary and Secondary school	44	10.0
	Vocational school	67	15.2
	Bachelor degree	280	63.6
	Master or PhD	49	11.1
Tenure	Less than 1 year	14	3.2
	1–5 year	139	31.6
	6–10 year	190	43.2
	10 and above	97	22.0

**Table 2:** Reliability and validity.

Variable	Item	Factor Loading	CICT	AVE	The Square Root of AVE	Cronbach’s α	CR
ESSL	ESSL1	0.837	0.816	0.604	0.777	0.947	0.948
	ESSL2	0.835	0.807				
	ESSL3	0.773	0.750				
	ESSL4	0.740	0.718				
	ESSL5	0.731	0.711				
	ESSL6	0.811	0.788				
	ESSL7	0.637	0.617				
	ESSL8	0.817	0.804				
	ESSL9	0.814	0.784				
	ESSL10	0.834	0.807				
	ESSL11	0.673	0.652				
	ESSL12	0.795	0.774				
GSE	GSE1	0.786	0.728	0.551	0.742	0.879	0.880
	GSE2	0.745	0.691				
	GSE3	0.796	0.753				
	GSE4	0.745	0.675				
	GSE5	0.697	0.640				
	GSE6	0.676	0.629				
GOI	GOI1	0.766	0.725	0.620	0.787	0.907	0.907
	GOI2	0.799	0.753				
	GOI3	0.796	0.746				
	GOI4	0.808	0.765				
	GOI5	0.755	0.719				
	GOI6	0.800	0.750				
EP	EP1	0.756	0.731	0.609	0.780	0.937	0.940
	EP2	0.752	0.720				
	EP3	0.813	0.787				
	EP4	0.764	0.734				
	EP5	0.759	0.738				
	EP6	0.789	0.760				
	EP7	0.810	0.786				
	EP8	0.753	0.719				
	EP9	0.805	0.784				
	EP10	0.799	0.768				
WPB	WPB1	0.752	0.715	0.581	0.762	0.906	0.906
	WPB2	0.758	0.723				
	WPB3	0.801	0.769				
	WPB4	0.683	0.651				
	WPB5	0.760	0.739				
	WPB6	0.801	0.742				
	WPB7	0.772	0.696				
GSV	GSV1	0.674	0.613	0.526	0.725	0.812	0.815
	GSV2	0.658	0.589				
	GSV3	0.732	0.637				
	GSV4	0.826	0.703				

Note: ESSL: Environmentally Specific Servant Leadership; WPB: Workplace Pro-environmental Behaviors; GSE: Green Self-Efficacy; GOI: Green Organizational Identity; EP: Environmental Passion; GSV: Green Shared Vision.

**Table 3:** Mean, standard deviation, and correlation.

	ESSL	GSE	GOI	EP	WPB	GSV	Gender	Age	Edu	Year
ESSL	1.000									
GSE	0.723 **	1.000								
GOI	0.695 **	0.727 **	1.000							
EP	0.739 **	0.641 **	0.641 **	1.000						
WPB	0.735 **	0.664 **	0.665 **	0.749 **	1.000					
GSV	0.346 **	0.189 **	0.288 **	0.234 **	0.247 **	1.000				
Gender	0.035	0.041	0.026	0.079	0.088	0.004	1.000			
Age	0.181 **	0.121 *	0.143 **	0.180 **	0.170 **	0.104 *	−0.112 *	1.000		
Edu	−0.098 *	−0.025	−0.054	−0.062	−0.055	−0.094 *	0.108 *	−0.290 **	1.000	
Tenure	0.225 **	0.196 **	0.174 **	0.172 **	0.228 **	0.164 **	−0.118 *	0.664 **	−0.208 **	1.000
Mean	3.807	4.012	3.876	3.993	4.102	4.177	1.450	2.210	2.760	2.840
SD	0.802	0.641	0.763	0.743	0.751	0.619	0.498	0.681	0.779	0.800

Note: ESSL: Environmentally specific Servant Leadership; WPB: Workplace Pro-environmental Behaviors; GSE: Green Self-efficacy; GOI: Green Organizational Identity; EP: Environmental Passion; GSV: Green Shared Vision; SD: Standard Deviation. * p < 0.05, ** p < 0.01.

**Table 4:** Results of regression analysis.

	GSE		GOI		EP		WPB							
	Model 1	Model 2	Model 3	Model 4	Model 5	Model 6	Model 7	Model 8	Model 9	Model 10	Model 11	Model 12	Model 13	Model 14
Gender	0.064	0.014	0.050	0.003	0.107 *	0.057	0.119 *	0.070 *	0.078 *	0.087 *	0.041	0.066 *	0.069 *	0.044
	(0.181)	(0.667)	(0.295)	(0.940)	(0.025)	(0.083)	(0.012)	(0.033)	(0.030)	(0.015)	(0.201)	(0.037)	(0.027)	(0.133)
Age	−0.012	−0.046	0.047	0.015	0.119	0.085	0.037	0.003	0.045	0.007	−0.050	0.016	−0.001	−0.035
	(0.857)	(0.314)	(0.462)	(0.755)	(0.063)	(0.055)	(0.557)	(0.939)	(0.356)	(0.886)	(0.247)	(0.711)	(0.982)	(0.380)
Edu	0.009	0.046	−0.015	0.021	−0.018	0.020	−0.012	0.025	−0.017	−0.002	0.001	0.013	0.019	0.016
	(0.862)	(0.186)	(0.760)	(0.568)	(0.715)	(0.553)	(0.806)	(0.457)	(0.638)	(0.950)	(0.972)	(0.697)	(0.554)	(0.594)
Tenure	0.213 **	0.076	0.145 *	0.013	0.102	−0.038	0.215 **	0.078	0.079	0.122 *	0.141 **	0.058	0.074	0.095 *
	(0.001)	(0.089)	(0.022)	(0.774)	(0.105)	(0.385)	(0.001)	(0.074)	(0.102)	(0.011)	(0.001)	(0.172)	(0.074)	(0.015)
ESSL		0.719 ***		0.691 ***		0.732 ***		0.716 ***				0.521 ***	0.513 ***	0.386 ***
		(0.000)		(0.000)		(0.000)		(0.000)				(0.000)	(0.000)	(0.000)
GSE									0.640 ***			0.272 ***		
									(0.000)			(0.000)		
GOI										0.640 ***			0.295 ***	
										(0.000)			(0.000)	
EP											0.731 ***			0.451 ***
											(0.000)			(0.000)
R2	0.043	0.529	0.034	0.483	0.049	0.553	0.066	0.549	0.458	0.462	0.575	0.584	0.594	0.640
Adj.R2	0.034	0.523	0.025	0.477	0.040	0.548	0.058	0.544	0.452	0.456	0.570	0.579	0.589	0.635
F	4.863 **	97.347 ***	3.844 **	81.218 ***	5.557 ***	107.380 ***	7.744 ***	105.866 ***	73.491 ***	74.596 ***	117.225 ***	101.442 ***	105.777 ***	128.562 ***
	(0.001)	(0.000)	(0.004)	(0.000)	(0.000)	(0.000)	(0.000)	(0.000)	(0.000)	(0.000)	(0.000)	(0.000)	(0.000)	(0.000)

Note: ESSL: Environmentally Specific Servant Leadership; WPB: Workplace Pro-environmental Behaviors; GSE: Green Self-Efficacy; GOI: Green Organizational Identity; EP: Environmental Passion; GSV: Green Shared Vision. * *p* < 0.05, ** *p* < 0.01, *** *p* < 0.001. The *p* values are in parentheses.

**Table 5:** The moderating effect of GSV.

	GSE		GOI		EP	
	Model 15	Model 16	Model 17	Model 18	Model 19	Model 20
Gender	0.015	0.014	0.002	0.001	0.057	0.055
	(0.655)	(0.680)	(0.949)	(0.967)	(0.082)	(0.084)
Age	−0.049	−0.048	0.017	0.017	0.084	0.084
	(0.281)	(0.276)	(0.719)	(0.717)	(0.058)	(0.053)
Edu	0.042	0.047	0.024	0.027	0.019	0.025
	(0.225)	(0.165)	(0.516)	(0.458)	(0.580)	(0.457)
Tenure	0.084	0.075	0.008	0.002	−0.035	−0.045
	(0.061)	(0.090)	(0.871)	(0.965)	(0.421)	(0.292)
ESSL	0.742 ***	0.710 ***	0.674 ***	0.654 ***	0.740 ***	0.705 ***
	(0.000)	(0.000)	(0.000)	(0.000)	(0.000)	(0.000)
GSV	−0.073 *	0.035	0.054	0.119 *	−0.024	0.094 *
	(0.040)	(0.444)	(0.146)	(0.013)	(0.482)	(0.032)
ESSL × GSV		0.156 ***		0.096 *		0.173 ***
		(0.000)		(0.032)		(0.000)
R2	0.533	0.548	0.486	0.491	0.554	0.571
Adj.R2	0.527	0.540	0.479	0.483	0.547	0.564
F	82.435 ***	74.722 ***	68.209 ***	59.612 ***	89.461 ***	82.221 ***
	(0.000)	(0.000)	(0.000)	(0.000)	(0.000)	(0.000)

Note: ESSL: Environmentally Specific Servant Leadership; WPB: Workplace Pro-environmental Behaviors; GSE: Green Self-Efficacy; GOI: Green Organizational Identity; EP: Environmental Passion; GSV: Green Shared Vision. * *p* < 0.05, *** *p* < 0.001. The *p* values are in parentheses.

## Data Availability

The original questionnaire data presented in this study are available on request from the corresponding author.

## Appendix A. Survey Items

**Table A1:** Measuring scale of variables.

Environmentally Specific Servant Leadership (ESSL)
ESSL is measured to what extent do you agree or disagree with the following statements on a 5-point Likert-scale (5-Strongly Agree, 1-Strongly Disagree)
ESSL1-My supervisor cares about my eco-initiatives.
ESSL2-My supervisor emphasizes the importance of contributing to the environmental improvement.
ESSL3-My supervisor is involved in environmental activities.
ESSL4-I am encouraged by my supervisor to volunteer in environmental activities.
ESSL5-My supervisor has a thorough understanding of our company and its environmental goals.
ESSL6-My supervisor encourages me to contribute eco-initiatives.
ESSL7-My supervisor gives me the freedom to handle environmental problems in the way that I feel is best.
ESSL8-My supervisor does what she/he can do to realize my eco-initiatives.
ESSL9-My supervisor holds high environmental standards.
ESSL10-My supervisor always displays green behaviors.
ESSL11-My supervisor would not compromise environmental principles in order to achieve success.
ESSL12-My supervisor values environmental performance more than profits.
Workplace Pro-environmental Behavior (WPB)
WPB is measured to what extent do you agree or disagree with the following statements on a 5-point Likert-scale (5-Strongly Agree, 1-Strongly Disagree)
WPB1-I print double sided whenever possible.
WPB2-I put compostable items in the compost bin.
WPB3-I put recyclable material (e.g., cans, paper, bottles, batteries) in the recycling bins.
WPB4-I bring reusable eating utensils to work (e.g., travel coffee mug, water bottle, reusable containers, reusable cutlery).
WPB5-I turn lights off when not in use.
WPB6-I take part in environmentally friendly programs (e.g., bike/walk to work day, bring your own local lunch day).
WPB7-I make suggestions about environmentally friendly practices to managers and/or environmental committees, in an effort to increase my organization’s environmental performance.
Green Self-efficacy (GSE)
GSE is measured to what extent do you agree or disagree with the following statements on a 5-point Likert-scale (5-Strongly Agree, 1-Strongly Disagree)
GSE1-We feel we can succeed in accomplishing environmental ideas.
GSE2-We can achieve most of environmental goals.
GSE3-We feel competent to deal effectively with environmental tasks.
GSE4-We can perform effectively on environmental missions.
GSE5-We can overcome environmental problems.
GSE6-We could find out creative solutions to environmental problems.
Green Organizational Identity (GOI)
GOI is measured to what extent do you agree or disagree with the following statements on a 5-point Likert-scale (5-Strongly Agree, 1-Strongly Disagree)
GOI1-The company’s top managers, middle managers, and employees have a strong sense of the company’s history about environmental management and protection.
GOI2-The company’s top managers, middle managers, and employees have a sense of pride in the company’s environmental goals and missions.
GOI3-The company’s top managers, middle managers, and employees feel that the company has carved out a significant position with respect to environmental management and protection.
GOI4-The company’s top managers, middle managers, and employees feel that the company have formulated a well-defined set of environmental goals and missions.
GOI5-The company’s top managers, middle managers, and employees are knowledgeable about the company’s environmental traditions and cultures.
GOI6-The company’s top managers, middle managers, and employees identify strongly with the company’s actions with respect to environmental management and protection.
Environmental Passion (EP)
EP is measured to what extent do you agree or disagree with the following statements on a 5-point Likert-scale (5-Strongly Agree, 1-Strongly Disagree)
EP1-I am passionate about the environment.
EP2-I enjoy practicing environmentally friendly behaviors.
EP3-I enjoy engaging in environmentally friendly behaviors.
EP4-I take pride in helping the environment.
EP5-I enthusiastically discuss environmental issues with others.
EP6-I get pleasure from taking care of the environment.
EP7-I passionately encourage others to be more environmentally responsible.
EP8-I am a volunteered member of an environmental group.
EP9-I have voluntarily donated time or money to help the environment in some way.
EP10-I feel strongly about my environmental values.
Green Shared Vision (GSV)
GSV is measured to what extent do you agree or disagree with the following statements on a 5-point Likert-scale (5-Strongly Agree, 1-Strongly Disagree)
GSV1-There is commonality of environmental goals in the company.
GSV2-There is total agreement on the company’s strategic environmental direction.
GSV3-All members in the company are committed to the environmental strategies of the company.
GSV4-The company’s employees are enthusiastic about the collective environmental mission of the company.
